# Concentrations of Persistent Organic Pollutants in Women’s Serum in the European Arctic Russia

**DOI:** 10.3390/toxics9010006

**Published:** 2021-01-07

**Authors:** Yulia Varakina, Dmitry Lahmanov, Andrey Aksenov, Anna Trofimova, Rimma Korobitsyna, Natalia Belova, Nikita Sobolev, Dmitry Kotsur, Tatiana Sorokina, Andrej M. Grjibovski, Valery Chashchin, Yngvar Thomassen

**Affiliations:** 1 Arctic Biomonitoring Laboratory, Northern (Arctic) Federal University Named after M. V. Lomonosov, Naberezhnaya Severnoy Dvini 17, 163002 Arkhangelsk, Russia; d.lahmanov@narfu.ru (D.L.); a.s.aksenov@narfu.ru (A.A.); a.trofimova@narfu.ru (A.T.); r.korobicina@narfu.ru (R.K.); belova-8@mail.ru (N.B.); n.sobolev@narfu.ru (N.S.); d.kocur@narfu.ru (D.K.); t.sorokina@narfu.ru (T.S.); valerych05@mail.ru (V.C.); yngvar.thomassen@stami.no (Y.T.); 2 Central Scientific Research Laboratory, Northern State Medical University of the Ministry of Healthcare of the Russian Federation, Troitskiy Ave. 51, 163000 Arkhangelsk, Russia; andrej.grjibovski@gmail.com; 3 N. Laverov Federal Center for Integrated Arctic Research, Ural Branch of the Russian Academy of Sciences, Naberezhnaya Severnoy Dvini 23, 163000 Arkhangelsk, Russia; 4 Department of Health Policy and Management, Al-Farabi Kazakh National University, Almay 050040, Kazakhstan; 5 Department of Epidemiology and Modern Vaccination Technologies, Sechenov First Moscow State Medical University (Sechenov University), 119991 Moscow, Russia; 6 West Kazakhstan Marat Ospanov Medical University, Aktobe 0300190, Kazakhstan; 7 North-Western State Medical University Named after I.I. Mechnikov, Kirochnaya ul. 41, 191015 Saint-Petersburg, Russia; 8 Institute of Ecology, National Research University Higher School of Economics, Myasnitskaya Str. 20, 101000 Moscow, Russia; 9 National Institute of Occupational Health, Gydas vei 8, N-0304 Oslo, Norway

**Keywords:** indigenous peoples, Russian Arctic, biomonitoring, persistent organic pollutants (POPs), PCB congeners, DDT, GC-MS/MS

## Abstract

Persistent organic pollutants (POPs) are heterogeneous carbon-based compounds that can seriously affect human health. The aim of this study was to measure serum concentrations of POPs in women residing in the Euro-Arctic Region of Russia. A total of 204 women from seven rural settlements of the Nenets Autonomous Okrug (NAO) took part in the study. We measured serum concentrations of 11 polychlorinated biphenyls (PCBs) and 17 organochlorine pesticides (OCPs) across the study sites and among Nenets and non-Nenets residents. Measurement of POPs was performed using an Agilent 7890A gas chromatograph equipped with an Agilent 7000 series MS/MS triple quadrupole system. The concentrations of all POPs were low and similar to findings from other Arctic countries. However, significant geographic differences between the settlements were observed with exceptionally high concentrations of PCBs in Varnek located on Vaygach Island. Both ΣDDT (*p* = 0.011) and ΣPCB (*p* = 0.038) concentrations were significantly lower in Nenets. Our main findings suggest that the serum concentrations of the legacy POPs in women in the Euro-Arctic Region of Russia are low and similar to those in other Arctic countries. Significant variations between settlements, and between Nenets and non-Nenets residents, were found. Arctic biomonitoring research in Russia should include studies on the associations between nutrition and concentrations of POPs.

## 1. Introduction

Persistent organic pollutants (POPs) are heterogeneous carbon-based compounds of natural or anthropogenic origin [1]. According to the Stockholm Convention on Persistent Organic Pollutants, 35 POPs are currently listed among the most harmful substances [2]. They persist over a long time in the environment, can be transmitted through food chains, and can accumulate in the tissues of living organisms causing a range of adverse effects on health [3,4,5]. Significant associations between POPs and common diseases, such as hormone-related cancers [1,6,7], type 2 diabetes [8], cardiovascular diseases [9], and Alzheimer’s disease [10], have been reported. Moreover, POPs are also associated with pregnancy complications [11], fetal and infant development [12], and secondary sex ratio [13]. However, the evidence is inconclusive and warrants further research.

The levels of POPs vary significantly between species and locations, and over time. Arctic ecosystems are particularly vulnerable to climate change and anthropogenic influences [14]. Bioaccumulation of POPs in food chains has a major impact on the health and well-being of people living in the far North, and particularly on indigenous health [15]. Human exposure to organochlorine pesticides (OCPs), such as dichlorodiphenyltrichloroethane (p,p′-DDT), hexachlorocyclohexanes (β-HCH), chlordanes, hexachlorobenzene (HCB), and mirex, has been a subject for extensive research in recent decades in the Arctic [16,17,18]. Although the concentrations of POPs in humans have decreased in most Arctic locations over the past two decades, the levels of oxychlordane, hexachlorobenzene (HCB), 2,2′,4,4′,5,5′-hexabromodiphenyl ether (PBDE153), and perfluorinated compounds (PFCs) remain stable [19].

The Russian Federation has sovereignty over one half of the Arctic, hosting two-thirds of the Arctic population [20], but information of POPs in international peer reviewed literature is limited. Research evidence on the concentration of POPs and their health effects for the residents of the Russian Arctic has been recently summarized elsewhere [19]. Similar to that of other Arctic settings, a decreasing trend in concentrations of oxychlordan, p,p′-DDT, p,p′-DDE, HCB, β-HCH, mirex, and ΣPCBs in maternal blood samples in Chukotka has been reported [21], but data from other parts of Arctic Russia is scarce. Moreover, the most recent monitoring studies in Chukotka were performed more than 5 years ago [22], warranting further research in other parts of Arctic Russia to provide up-to-date information about POPs in the high North.

Earlier we identified the challenges associated with insufficient integration of Russia with global monitoring of POPs, proposed changes to harmonize monitoring activities in Russia aiming at filling the gaps in the Arctic Monitoring and Assessment Programme (AMAP) [23], and contributed with an assessment of POPs in fish in the Nenets Autonomous Okrug (NAO) in the European region of the Russian Arctic [24].

The aim of this study was to assess concentrations of selected POPs among women in NAO with special emphasis on ethnic background and place of residence.

## 2. Materials and Methods

### 2.1. Study Design and Sampling

**Figure 1:**
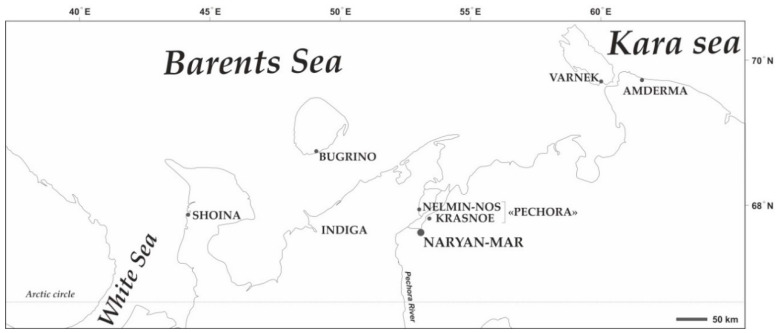
Location of the study settlements. The map was created by Andrey S. Aksenov using CorelDRAW Graphics Suite X4 software (license certificate № 30064931). (https://www.coreldraw.com/); the topographic base of the map was created with Natural Earth Free Vector and Raster Map Data (https://www.naturalearthdata.com).

NAO is one of the northernmost and least populated (44.1 thousand in 2020) federal subjects of the Russian Federation. The regional capital of Naryan-Mar accounts for 51.4% of NAO’s population. Most settlements in NAO have little connection with the regional center for most of the year. NAO has a sub-Arctic climate with long and cold winters and short and wet summers, strong winds, and frequent weather changes. Russians (63.3%) and Nenets (17.8%) are the main ethnic groups. Reindeer herding and fishing are the main sources of income in the rural areas, although oil and gas industry is also present in the area. The study was performed from July to October 2018 in 7 rural settlements in NAO, namely, Bugrino, Varnek, Amderma, Indiga, Shoina, Nelmin-Nos, and Krasnoe (Figure 1). The data from Nelmin-Nos and Krasnoe were merged with that of Pechora due to similarities between these two settlements situated along the Pechora river.

All adult residents of the abovementioned settlements were informed about the study and invited to participate by the local health care units. Among those who participated, we selected only those who lived at least half of their lives in the index settlement. After applying exclusion criteria [25] and excluding those with acute illnesses and pregnant and lactating women, the final sample consisted of 204 women. The participation rate ranged from 5.2% of the total female population in Pechora to 22% in Varnek. All participants filled out a questionnaire with the assistance of trained local health care workers. Fasting blood samples were taken the same day [25]. By ethnicity, the participants were divided into Nenets (*n* = 113) representing the indigenous population and non-Nenets (*n* = 91) consisting of mostly Russians permanently living in NAO. Body mass index (BMI) was calculated for all participants. Overweight and obesity were defined as 25.0 < BMI < 29.9 kg/m^2^ and BMI > 30.0 kg/m^2^, respectively. More details on the settings and recruitment of the participants, in addition to blood and serum sample collection, transportation, and storage are presented elsewhere [25].

### 2.2. Materials

The concentrations of chloroorganic pesticides and their metabolites, namely, α-HCH, β-HCH, γ-HCH, p,p′-DDE, p,p′-DDD, o,p′-DDE, o,p′-DDD, heptachlor, cis-chlordane, trans-chlordane, cis-nonachlor, trans-nonachlor, aldrin, mirex, hexachlorobenzene, 1,2,3,5-tetrachlorobenzene, and 1,2,4,5-tetrachlorobenzene, and the polychlorinated biphenyls (PCBs) 28, 52, 101, 105, 118, 123, 128, 138, 153, 180, and 183, were measured in serum.

HPLC-grade hexane and all high-purity analyte standards were obtained from Sigma-Aldrich (Steinheim, Germany). Labeled ^13^C_12_ PCB101 was obtained from Cambridge Isotope Laboratories (Tewksbury, MA. USA) and 2,4,5,6-tetrachloro-m-xylene, 4,4′-dibromooctafluorobiphenyl, and 2,3,5,6-Tetrabromo-p-xylene (TBX) were obtained from Sigma-Aldrich (Steinheim, Germany). Sulfuric acid (98% purity) was purchased from Supelco (Bellefonte, PA. USA). Helium (99.9999% purity) and nitrogen (99.9999% purity) were obtained from NIIKM (Moscow, Russia).

### 2.3. Serum Analysis

Before extraction, frozen blood serum samples were kept at 4 °C for 24 h. Two mL portion of serum sample was placed into a 15 mL plastic centrifuge tube and 20 µL of an internal standards mixture (2,4,5,6-tetrachloro-m-xylene, 4,4′-dibromooctafluorobiphenyl, 2,3,5,6-Tetrabromo-p-xylene (TBX), PCB101^13^C_12_ was added. Then, 3 mL of conc. H_2_SO_4_ and 3 mL of n-hexane were added. The resulting suspension was vigorously shaken for 30 s and centrifuged (1500 rpm, 10 min).

The supernatant of the n-hexane layer was transferred to a second 15 mL centrifuge tube. Then, 2 mL of n-hexane was added to the first tube containing H_2_SO_4_ and plasma, mixed and centrifuged as described above; this step was repeated twice.

To the resulting 7 mL supernatant volume, 2 mL of conc. H_2_SO_4_ was added before mixing and centrifugation, as described above. The final supernatant was transferred to a glass conical bottom centrifuge tube and evaporated to dryness in a vacuum rotary evaporator EV400 LabTech (Italy) at 53 °C. Then, the sample was transferred to the micro vials with two portions of acetone in 50 µL each. If extraction emulsions formed at any stage, they were eliminated by adding 10–15 drops of MilliQ water before centrifugation [26].

### 2.4. Instrumental Analysis

An Agilent 7890A gas chromatograph (GC) with an Agilent 7000 series MS/MS triple quadrupole system (Santa Clara, CA, USA) worked in electron-ionization (EI) mode (70 eV) was used as the main equipment. An Agilent ultra-inert GC column (HP-5MS UI, 30 m × 0.25 mm × 0.25 µm) with a Restek Guard column (Rtx-5M) was used for analysis.

A multimode injector was operated in splitless mode at a temperature of 250 °C. The portions of extracts were 1 µL. The ultra-inlet liner was with a glass wool frit 5190-2293 from Agilent (Santa Clara, CA. USA).

GC MS/MS settings were the same as in the article [24].

### 2.5. Quality Assurance, Quality Control and Method Validation

Three replicate test portions of the 5 serum samples were weighed into 15 mL tubes.

For validation, a blank matrix and matrix spiked at 0.2 and 1 ng/mL were used (Table 1). Blank matrix was obtained by mixing together equal parts of different blood and was used in matrix-matched (MM) calibration at 0, 2, 5, 20, 40, and 80 ng/mL levels. Daily validation included a reagent blank analysis. In real sample analysis, each batch (10 samples) included the hexane blank and reagent sample blank.

Limit of detection (LOD) was calculated as three times the standard deviation of the procedural blank levels. Limit of quantification (LOQ) was calculated as ten times the standard deviation of the procedural blank levels. If the signal was not detected, then serum with a minimal addition of analytes was used to determine LOD and LOQ (Table 1). If values were below the LOD, we assigned the values of ½ LOD.

Blank and calibration samples were prepared daily. Linear regression coefficients were ≥0.99 and all relative standard deviations (RSD) were below 20%. For assessing the accuracy of obtained results, a spiked mixture of serum blood with known content of analytes (0.5 ng/mL) was analyzed in each batch. Average batch accuracy is presented Table 1.

### 2.6. Determination of Total Serum Lipids

All POP concentrations were adjusted for serum lipids. The total lipid content (mg/dL) was calculated from the concentrations of total cholesterol and triglycerides according to the following equation [27,28]:

Total lipids = 2.27 ∗ total cholesterol + triglycerides + 62.3

Total cholesterol and triglyceride were assessed by enzymatic methods using an automatic biochemical analyzer Random Access A-15 (Biosystems, Barcelona, Spain) at the Northern State Medical University (Arkhangelsk).

### 2.7. Statistical Analysis

To ensure comparability of our results with the findings of other studies we used means, medians, and geometric means (GM) as measures of central tendency and standard deviations and range as measures of variability. In addition, 95% confidence intervals (CI) were calculated for GM. Distributions of continuous variables were assessed using Shapiro–Wilk tests. Given that most of the variables were skewed to the right, non-parametric statistical tests were applied for all analyses. Kruskal-Wallis tests were used for analyses across the settings, and differences between Nenets and non-Nenets were studied by Mann–Whitney tests. Categorical variables were compared using Pearson’s chi-squared tests. IBM SPSS software, version 23.0 (IBM Corp., Armunk, NY, USA) was used for all calculations.

### 2.8. Ethical Considerations

The study was approved by the Ethical Committee at the Northern Medical State University, Arkhangelsk, Russia (protocol no. 06/09-17 of 27 September 2017). All study participants signed an informed consent.

## 3. Results

### 3.1. Basic Characteristics of the Study Participants

The age of the women ranged from 19 to 87 years. Nenets constituted 55.4% of the sample. Every fourth woman was a smoker with no differences across the settlements (*p* = 0.253) or ethnic background (*p* = 0.935). The participants’ age, BMI, total cholesterol, triglycerides, total serum lipids and proportion of smokers across study sites and ethnic backgrounds is presented in Table 2. Only age and ethnic background varied significantly between the settlements Nenets women were significantly younger (*p* < 0.001) and tended to have lower concentrations of triglycerides (*p* = 0.053) and total lipids (*p* = 0.058). As many as 66.0% of the women were overweight (40.4%) or obese (25.6%) with no significant ethnic differences.

### 3.2. Serum Concentrations of Lipophilic PCB

The following serum POPs were detected in women: PCB 118, PCB 138, PCB 153, PCB 180, PCB 183, p,p′-DDE, o,p′-DDE, p,p′-DDD, hexachlorobenzene (HCB), β-hexachlorocyclohexane (β-HCH), aldrin, and mirex. Other compounds, such as PCB 28, 52, 101, 105, 123, 128, α-HCH, γ-HCH, heptachlor, 1,2,3,5-tetrachlorobenzene, 1,2,4,5-tetrachlorobenzene, cis-chlordane, trans-chlordane, cis-nonachlor and trans-nonachlor, were below LOQ. Congeners 118, 138 and 153, in addition to o,p′-DDE, p,p′-DDE, HCB, and β-HCB, were found in 100% of the samples. The detection rate ranged from 5.4 to 66.2% for other compounds.

#### 3.2.1. Distribution of POPs across the Study Settlements

The concentrations of POPs across the settlements are presented in Table 3.

Plasma concentrations of PCB 118 (*p* < 0.001), PCB 138 (*p* < 0.001), PCB 153 (*p* < 0.001), PCB 180 (*p* < 0.001), and PCB 183 (*p* = 0.002) varied significantly between the settlements. As 95% CIs for GM indicate, women living in Varnek had the highest concentrations of PCB 153, 180, and 183. Significantly higher PCB 118 concentrations were observed in Amderma, whereas women from Shoina had higher concentrations of PCB 138.

Among the organochlorine pesticides (OCPs), p,p′-DDE showed the highest concentrations among the participants from all settlements with a median of 68.8 ng/g lipid. Regarding PCBs, significant variations in concentrations of OCPs between the settlements were observed.

**Figure 2:**
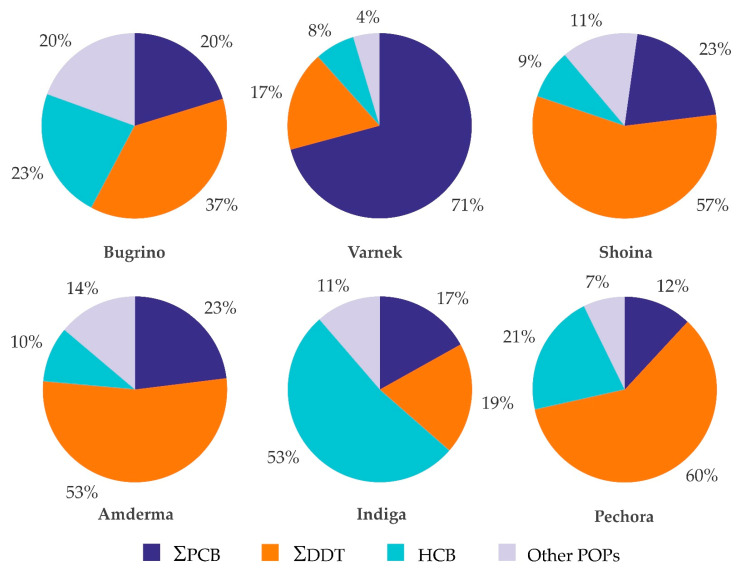
Proportions of selected persistent organic pollutants (POPs) of the total POP concentration in serum of women living in different settlements of the Nenets Autonomous Okrug (Arctic Russia).

ΣDDT was the dominant POP in all settlements, except Varnek and Indiga, where ΣPCB and HCB, respectively, were the dominant compounds. The main congener of the ΣDDT group was p,p′-DDE. The group “others” included β-HCH, aldrin, and mirex (Figure 2).

#### 3.2.2. Distribution of POPs between Nenets and Non-Nenets

**Figure 3:**
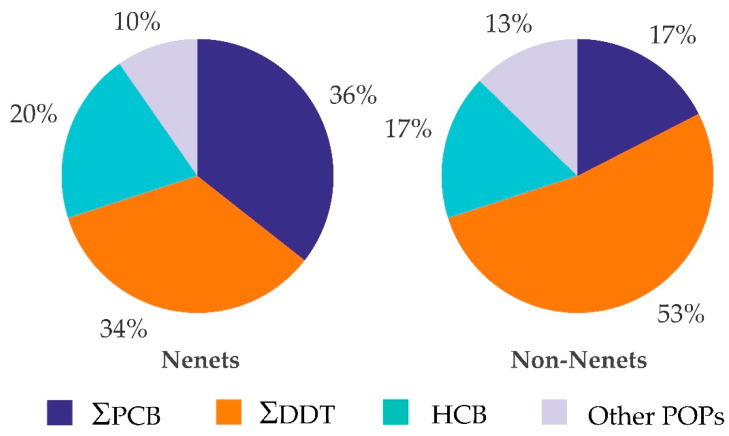
Contribution of selected pollutants to the total serum POP concentration among Nenets and non-Nenets women.

Nenets women had higher concentration of PCB 183 (*p* = 0.013), but lower concentration of PCB 118 (*p* = 0.001). Concentration of o,p′-DDE was greater in Nenets (*p* < 0.001), whereas the concentration of p,p′-DDE was greater among non-Nenets (*p* = 0.006). Both ΣDDT (*p* = 0.011) and ΣPCB (*p* = 0.038) concentrations were significantly lower in Nenets. However, ΣDDT comprised 34% of the measured POPs in the serum of Nenets but 53% of non-Nenets, whereas ΣPCB comprised 36% of POPs in Nenets but 17% in non-Nenets (Figure 3).

## 4. Discussion

To the authors’ knowledge this is the largest study on the concentration of POPs in women’s serum conducted in the European part of the Russian Arctic. Most previous studies were performed in a limited number of settlements. Studies from Greenland and Northern Canada are exceptions because POP biomonitoring is regulated by the state [19,29,30,31].

Geometric means for ∑PCB_11_ and ∑DDT were 58.5 ng/g lipid and 136 ng/g lipid, respectively, which is similar to that reported in international literature [22,32,33,34]. Geometric means of marker polychlorinated biphenyls (∑PCB_6_) were 37.9 ng/g lipid. Increased concentrations of PCB 153 were observed in Varnek, located on Vaygach Island, p,p′-DDE in the west (Shoina), PCB 138 in the east (Amderma), and HCB in the central part of NAO (Indiga, Nelmin-Nos, Krasnoe). Concentrations of POPs in Arctic residents have been shown to be associated with traditional food items rich in fat [16,17,22,29]. The differences on serum concentrations of POPs observed in this study may be partly attributed to the differences between the settlements in food patterns [35], although the historic use of DDT as an insecticide in the studied settlements may also be an important factor. Further research is needed to elucidate the reasons behind the observed differences.

**Figure 4:**
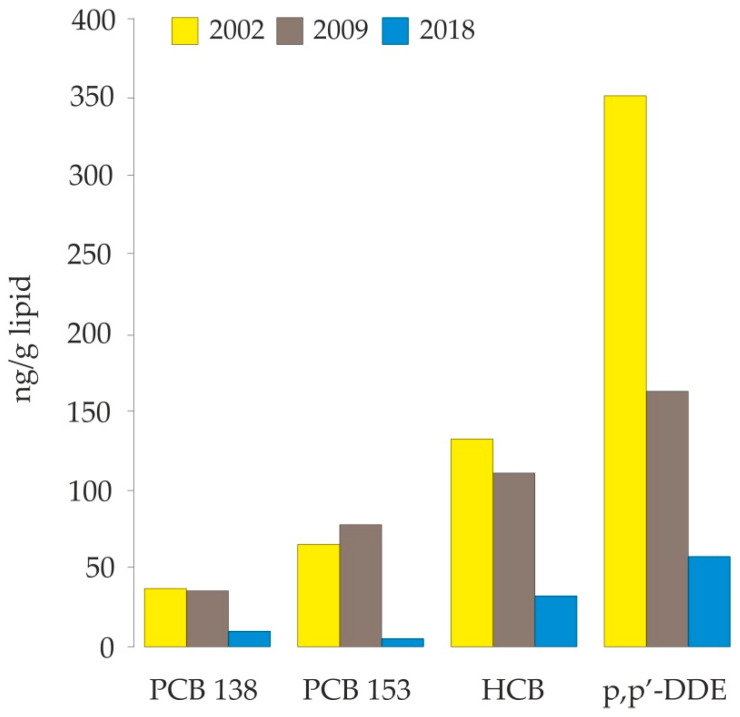
Concentration of selected POPs (geometric means) in serum of women from 2002 to 2018 in Nelmin-Nos, Nenets Autonomous Okrug (Russia Arctic).

Concentrations of most POPs in human serum have decreased in recent decades [19,22]. In the European part of the Russian Arctic, temporal trends are only available for Nelmin-Nos. Our results indicate a clear downward trend in serum concentrations of p,p′-DDE, HCB, PCB 138, and PCB 153 from 2002 to 2009 [36] and further to 2018 (Figure 4). Interestingly, the most pronounced decline was observed between 2009 and 2018.

The downward trend in concentrations of POPs has also been reported in other countries (Table 4). For example, the concentration of p,p′-DDE and PCB153 in the serum of women in Greenland halved, the concentration of HCB decreased six times, and the concentration of β-HCH decreased 10 times in the period from 2005 to 2015 [29,30,31]. The most likely explanation for this is prohibition of the use of POPs.

Higher average concentrations and maximum values for PCB 153 (2413 ng/g lipid) and p,p′-DDE (300 ng/g lipid) concentrations in Varnek may be associated with the increased frequency in dietary consumption of marine mammals, which accumulate these substances. Associations between location-specific food patterns and concentrations of POPs and other environmental pollutants in residents of the European Arctic Russia are a subject for our future research.

Significant variations in concentrations of selected POPs between the settlements suggest that national or regional data can mask local geographical differences, warranting more detailed biomonitoring activities.

Although our study presents unique findings of different POPs in women’s serum in European Arctic Russia, the results should be interpreted with caution taking into account potential limitations of the study. In spite the fact that all residents of the selected settlements had an opportunity to take part in the study, one may suspect that those with poorer health were more likely to contact healthcare facilities for participation. If we hypothesize that health is inversely associated with exposure to POPs, this situation may have led to overestimation of the average values of POPs in this study. This concern is partly supported by the observed suboptimal average levels of cholesterol and triglycerides, although the prevalence of obesity is comparable with the national average [25].

Although we collected the data from seven rural settlements, they may not be representative of NAO, where 73% of inhabitants live in towns. However, they were selected from east, west, and central NAO, and the islands, for better representativeness of the rural part of NAO. Nevertheless, given that the majority of the population live in urban areas and the regional capital Naryan-Mar, which is home to more than a half of the population of NAO, these residents should also be included in biomonitoring activities.

We emphasize that biomonitoring studies should be carried out regularly in selected locations to obtain the most accurate picture of the concentrations of POPs in humans and their trends. Because the ban on the use of POPs is regulated by the Stockholm Convention, the main remaining sources of legacy POPs in the Arctic appears to be local food. Since 2017, the Arctic biomonitoring laboratory at NArFU (Arkhangelsk) has been collecting food samples among the population of NAO.

## 5. Conclusions

Our findings suggest that the concentrations of most POPs in women’s serum in NAO of European Arctic Russia are low and similar to those in other Arctic countries. Significant geographical variations between settlements, and between Nenets and non-Nenets in the study area were found. Arctic biomonitoring research in Russia should include studies of associations between nutrition and concentrations of POPs.

## Figures and Tables

**Table 1:** Analyte recoveries.

Analyte	LOD	LOQ	Spiked Level				Accuracy (%)
			0.2 ng/mL		1 ng/mL		
	ng/mL		Recovery (%)	RSD (%)	Recovery (%)	RSD (%)	
1,2,3,5-Tetrachlorobenzene	0.01	0.04	106	9	102	8%	112
1,2,4,5-Tetrachlorobenzene	0.01	0.04	106	9	102	8%	112
α-HCH	0.06	0.21	126	8	110	7%	96
HCB	0.03	0.11	126	12	97	7%	102
β-HCH	0.02	0.07	118	13	98	8	93
γ-HCH	0.04	0.13	121	5	110	7	101
PCB28	0.004	0.02	119	10	105	5	90
Heptachlor	0.09	0.29	118	12	115	9	120
PCB52	0.03	0.11	105	5	99	6	90
Aldrin	0.09	0.24	75	20	86	8	120
o,p′-DDE	0.02	0.07	123	11	90	9	112
trans-Chlordane	0.1	0.32	117	14	102	7	86
PCB101	0.05	0.15	102	8	94	5	92
cis-Chlordane	0.11	0.38	109	17	111	8	120
trans-Nonachlor	0.09	0.29	105	14	86	7	101
p,p′-DDE	0.11	0.36	108	10	114	15	98
o,p′-DDD	0.01	0.03	129	10	111	5	104
PCB123	0.05	0.16	116	7	98	5	95
PCB118	0.08	0.27	124	11	109	7	103
p,p′-DDD	0.05	0.16	113	7	94	8	100
cis-Nonachlor	0.04	0.13	124	5	101	8	111
PCB153	0.02	0.08	126	15	106	6	90
PCB105	0.07	0.22	117	9	97	4	96
PCB138	0.09	0.31	121	13	108	5	90
PCB183	0.05	0.18	89	10	88	7	90
PCB128	0.05	0.16	109	7	105	18	93
PCB180	0.01	0.04	106	11	97	7	103
Mirex	0.06	0.2	77	13	85	6	104

**Table 2:** Characteristics of the study population: Women in Nenets Autonomous Region.

Characteristic	Measures	Bugrino	Varnek	Amderma	Shoina	Indiga	Pechora	*p*-Value ^1^	Nenets	Non-Nenets
Age (years)	*n* (%)	24(11.8)	11(5.4)	33(16.2)	32(15.7)	35(17.1)	69(33.8)		113 (55.4)	91 (44.6)
	Mean (SD)	41.9 (11.3)	46.6 (15.5)	49.1(12.5)	49.7 (16.2)	59.9 (15.1)	49.2 (12.8)	<0.001	46.6 (13.9)	54.5 (14.1)
	Median Range	43 20–59	52 22–65	53 28–69	53 24–87	61 19–86	49 24–71		46 20–79	55 19–87
Proportion of Nenets	%	22 (91.6)	11 (100)	11 (33.3)	11 (34.4)	15 (42.8)	43 (62.3)			
BMI (kg/m^2^) ^2^	Mean (SD)	27.8 (6.9)	25.7 (6.2)	30.0 (7.0)	27.1 (4.9)	26.8 (3.5)	27.5 (5.3)	0.426	27.1 (5.8)	28.4 (5.4)
	Median Range	26.4 17.1–41.4	24.4 16.4–39.6	29.3 20.9–46.0	26.9 16.2–42.7	26.7 19.9–35.1	26.9 17.0–42.8		26.6 16.2–42.9	27.3 19.2–46.0
Cholesterol (mmol/L)	Mean (SD)	5.8 (1.1)	5.0 (1.3)	5.7 (1.2)	5.7 (1.4)	5.3 (0.9)	5.8 (1.4)	0.140	5.5 (1.1)	5.9 (1.2)
	Median Range	5.4 4.3–8.1	5.0 2.8–7.4	5.5 2.7–7.7	5.6 3.7–10.6	5.2 3.1–7.2	5.9 3.9–8.0		5.4 2.8–8.0	5.6 3.8–10.6
Triglycerides (mmol/L)	Mean (SD)	1.4 (0.8)	2.1 (1.6)	1.9 (1.1)	1.5 (0.7)	1.4 (0.8)	1.4 (0.7)	0.267	1.5 (0.9)	1.6 (0.8)
	Median Range	1.0 0.4–3.6	1.7 0.4–5.5	1.8 0.3–4.3	1.4 0.2–2.9	1.4 0.5–4.1	1.4 0.4–2.9		1.4 0.2–5.5	1.5 0.3–4.3
Total Lipid (mg/dL)	Mean (SD)	618 (116)	583 (138)	638 (127)	623 (141)	581(106)	628 (113)	0.374	601 (122)	637 (119)
	Median Range	576 453–854	565 371–802	647 315–856	620 408–1079	559 353–801	640 425–852		588 315–856	629 444–1079
Smoking	Yes (%)	4 (16.7)	3 (27.3)	10 (30.3)	11 (34.4)	4 (11.4)	19 (27.5)		28(24.8)	23(25.3)

^1^*p*-value for difference between the settings calculated using Kruskal–Wallis test. ^2^ BMI: Body mass index, calculated as kg/m^2^.

**Table 3:** Serum concentrations of polychlorinated biphenyls (PCBs) and organochlorine (OC) pesticides.

Analyte	Measures	Bugrino	Varnek	Shoina	Amderma	Indiga	Pechora	Nenets	Non-Nenets
		(*n* = 24)	(*n* = 11)	(*n* = 32)	(*n* = 33)	(*n* = 35)	(*n* = 69)	(*n* = 113)	(*n* = 91)
**PCBs (ng/g lipid)**									
PCB118	GM (95% CI)	8.2 (6.7–10.0)	12.2 (5.5–24.5)	11.0 (8.2–14.9)	18.2 (14.9–22.2)	11.0 (8.2–14.9)	9.0 (8.2–11.0)	9.0 (8.2–11.0)	13.5 (12.2–16.4)
	Median	7.3	7.3	7.9	18.3	7.5	7.8	7.5	12.0
	Range	<LOD–34.6	<LOD–183	<LOD–76.9	<LOD–48.2	<LOD–128	<LOD–57.6	<LOD–183	<LOD–118
PCB138	GM (95% CI)	12.2 (8.2–16.4)	12.2 (8.2–16.4)	90.0 (30.0–244)	18.2 (13.5–22.2)	16.4 (12.2–22.2)	10.0 (8.2–13.5)	13.5 (11.0–16.4)	13.5 (11.0–14.9)
	Median	8.6	143	17.7	16.0	8.2	7.9	9.0	9.8
	Range	<LOD–116	<LOD–811	<LOD–114	<LOD–67.7	<LOD–144	<LOD–96.9	<LOD–811	<LOD–114
PCB153	GM (95% CI)	14.9 (8.2–27.1)	200 (44.7–897)	18.2 (12.2–30.0)	24.5 (16.4–36.6)	8.2 (5.0–13.5)	8.2 (6.0–12.2)	12.2 (9.0–18.2)	16.4 (13.5–22.2)
	Median	19.5	558	23.7	31.1	10.8	9.5	13.8
	Range	<LOD–260	<LOD–2413	<LOD–171	<LOD–196	<LOD–246	<LOD–164	<LOD–2413	<LOD–171
PCB180	GM (95% CI)	9.0 (6.7–13.5)	121 (33.1–445)	7.4 (5.0–11.0)	11.0 (6.7–16.4)	2.2 (1.5–3.7)	<LOD	4.5 (3.0–6.0)	3.0 (2.5–4.1)
	Median	7.3	298	8.6	13.2	1.1		5.0	2.7
	Range	<LOD–270	<LOD–1006	LOD–74.6	LOD–96.6	LOD–77.8		<LOD–1006	<LOD–61.7
PCB183	GM (95% CI)	<LOD	13.5 (7.4–27.1)	<LOD	<LOD	<LOD	<LOD	5.0 (4.5–5.0)	4.1 (3.7–4.1)
	Median		17.7					4.3	4.0
	Range		<LOD–48.5					<LOD–48.6	<LOD–5.6
**OC Pesticides (ng/g lipid)**									
o,p′-DDE	GM (95% CI)	16.4 (11.0–27.1)	24.5 (16.4–36.6)	12.2 (9.0–18.2)	5.0 (3.0–8.2)	3.3 (2.5–4.5)	33.1 (24.5–44.7)	16.4 (12.2–20.1)	9.0 (7.4–12.2)
	Median	22.4	29.0	15.1	2.0	2.1	41.6	23.1	14.5
	Range	<LOD–77.0	8.7–67.0	<LOD–243	<LOD–275	<LOD–19.7	<LOD–596	<LOD–597	<LOD–144
p,p′-DDE	GM (95% CI)	73.7 (49.4–121)	99.5 (30.0–330)	110 (66.7–164)	148 (109–200)	40.4 (27.1–54.6)	60.3 (49.4–81.5)	11.0 (9.0–14.9)	109.6 (81.5–134)
	Median	60.5	155	121	142	37.9	65.3	59.9	125.3
	Range	<LOD–733	<LOD–1239	<LOD–1318	28.0–1313	LOD–336	<LOD–2577	<LOD–1313	<LOD–2577
p,p′-DDD	GM (95% CI)	<LOD	<LOD	<LOD	<LOD	<LOD	54.6 (49.4–66.7)	54.6 (40.4–66.7)	9.0 (7.4–12.2)
	Median						35.7	5.3	4.5
	Range						<LOD–194	<LOD–194	<LOD–152
HCB	GM (95% CI)	49.4 (27.1–90.0)	73.7 (27.1–181)	20.1 (13.5–30.0)	33.1 (24.5–44.7)	99.5 (54.6–164)	54.6 (44.7–73.7)	12.2 (10.0–16.4)	44.7 (33.1–54.6)
	Median	68.7	77.9	26.2	39.3	150	71.2	74.3	42.1
	Range	<LOD–255	<LOD–363	<LOD–121	<LOD–114	<LOD–767	<LOD–335	<LOD–767	<LOD–723
β-HCH	GM (95% CI)	10.0 (5.5–16.4)	16.4 (7.4–36.6)	22.2 (13.5–33.1)	33.1 (22.2–44.7)	20.1 (14.9–30.0)	14.9 (11.4–20.1)	9.0 (8.2–11.0)	27.1 (22.2–36.6)
	Median	9.1	17.8	25.0	41.5	19.0	16.5	13.0	32.0
	Range	<LOD–136	<LOD–81.5	<LOD–275	7.3–232	<LOD–123	<LOD–313	<LOD–34.0	<LOD–55.7
Aldrin	GM (95% CI)	20.1 (11.0–36.6)	12.2 (5.5–27.1)	<LOD	8.2 (6.7–10.0)	<LOD	<LOD	5.0 (4.5–5.5)	8.2 (7.4–9.0)
	Median	8.2	8.2		7.3			7.8	7.3
	Range	<LOD–351	<LOD–352		<LOD–50.5			<LOD–352	<LOD–103
Mirex	GM (95% CI)	<LOD	5.5 (4.1–6.7)	<LOD	<LOD	5.5 (4.1–6.7)	<LOD	5.0 (4.5–5.5)	4.5 (4.1–5.0)
	Median		5.5			5.2		5.0	4.7
	Range		<LOD–10.0			<LOD–111		<LOD–111	<LOD–6.6

**Table 4:** Geometric means for selected POPs in women’s serum, ng/g lipid.

Location (year)	p,p′-DDE	PCB 118	PCB 138	PCB 153	HCB	β-HCH	Reference
Arctic Russia							
NAO (2018)	73.7	11.0	13.5	14.9	49.4	18.2	Present study
Nelmin-Nos (2018)	59.5	9.0	9.0	6.3	33.8	35.7	
Nelmin-Nos (2002–2003)	246	48.0	46.0	98.0	135		[32]
Chukotka (2001–2002)	308			97.0			[33]
Chukotka (2015)	120	10.0	17.0	31.0	35.0	35.0	[22]
Other Arctic locations							
Greenland (2010–2013)	130	9.4	29.5	60.5	24.5	3.8	[29]
Greenland (2010–2015)	120	8.4	26.0	53.0	26.0	3.6	[30]
Greenland (1999–2005)	238			104	152	37.8	[31]
Norway (2004)	38.7			24.8			[37]

## Data Availability

The data presented in this study are available on request from the corresponding author.
